# Untargeted Metabolomics Study of the In Vitro Anti-Hepatoma Effect of Saikosaponin d in Combination with NRP-1 Knockdown

**DOI:** 10.3390/molecules24071423

**Published:** 2019-04-11

**Authors:** Yingtong Lv, Xiaoying Hou, Qianqian Zhang, Ruiting Li, Lei Xu, Yadong Chen, Yuan Tian, Rong Sun, Zunjian Zhang, Fengguo Xu

**Affiliations:** 1 Key Laboratory of Drug Quality Control and Pharmacovigilance (Ministry of Education), State Key Laboratory of Natural Medicine, China Pharmaceutical University, Nanjing 210009, China; lv_yingtong@126.com (Y.L.); cpuhxy_2011@163.com (X.H.); zhangqianqian09413@163.com (Q.Z.); 15051853218@163.com (R.L.); 18356002797@163.com (L.X.); 1020041334@cpu.edu.cn (Y.T.); 2 Jiangsu Key Laboratory of Drug Screening, China Pharmaceutical University, Nanjing 210009, China; 3 Department of Organic Chemistry, China Pharmaceutical University, Nanjing 210009, China; ydchen@cpu.edu.cn; 4 Advanced Medical Research Institute, Shandong University, Jinan 250100, China; sunrong107@163.com

**Keywords:** saikosaponin d, neuropilin-1, HepG2, metabolomics, metabolite deregulation score

## Abstract

Saikosaponin d (SSd) is one of the main active ingredients in Radix Bupleuri. In our study, network pharmacology databases and metabolomics were used in combination to explore the new targets and reveal the in-depth mechanism of SSd. A total of 35 potential targets were chosen through database searching (HIT and TCMID), literature mining, or chemical similarity predicting (Pubchem). Out of these obtained targets, Neuropilin-1 (NRP-1) was selected for further research based on the degree of molecular docking scores and novelty. Cell viability and wound healing assays demonstrated that SSd combined with NRP-1 knockdown could significantly enhance the damage of HepG2. Metabolomics analysis was then performed to explore the underlying mechanism. The overall difference between groups was quantitatively evaluated by the metabolite deregulation score (MDS). Results showed that NRP-1 knockdown exhibited the lowest MDS, which demonstrated that the metabolic profile experienced the slightest interference. However, SSd alone, or NRP-1 knockdown in combination with SSd, were both significantly influenced. Differential metabolites mainly involved short- or long-chain carnitines and phospholipids. Further metabolic pathway analysis revealed that disturbed lipid transportation and phospholipid metabolism probably contributed to the enhanced anti-hepatoma effect by NRP-1 knockdown in combination with SSd. Taken together, in this study, we provided possible interaction mechanisms between SSd and its predicted target NRP-1.

## 1. Introduction

Radix Bupleuri is a common traditional Chinese medicine (TCM), which has been used in China for over 2000 years. Saikosaponin d (SSd), as one of the main active ingredients extracted from Radix Bupleuri, has been proved to be liver-protective and is used to treat liver fibrogenesis [1], inflammation [2], and viral hepatitis. It was also reported that SSd exerted antitumor [3], immunoregulation [4], neuroregulation [5], and anti-allergic activities [6]. However, on the contrary, studies showed that SSd also had toxicity and could induce liver injury [7], hepatocyte or hepatic stellate cells apoptosis [8,9]. The effect of SSd is diverse and the underlying mechanisms remain unclear.

Target predicting is a feasible strategy that could facilitate a mechanistic study of TCM. For TCM, databases such as HIT, TCMSP, TCM Database@Taiwan, and TCMID can be used to search for potential targets [10,11,12]. However, databases cannot update reported targets. On the other hand, different databases contain different targets due to various algorithms. There is no database integrating targets from all sources yet. To further explore unreported targets, predicting approaches based on chemoinformatics could be applied, such as ligand-based prediction, receptor-based prediction, and data mining-based prediction [13,14,15,16]. However, the three-dimensional structures of targets are usually ignored, so the mutual binding mode of drug and targets cannot be fully reflected. Thus, for the targets obtained, molecular docking can be applied to evaluate the binding affinity between SSd and proteins, which is a fast, accurate method to predict structural affinity.

NRP-1 is a transmembrane glycoprotein composed of five domains (a1, a2, b1, b2, c) [17], whereas a1/a2 subunit mainly binds class-3 semaphorins and transduces signals by conjunction with plexins [18]. Vascular endothelial growth factors (VEGF) interact with b1/b2 subunit to exert angiogenesis regulation effects [19]. Studies have shown that NRP-1 was upregulated in liver cancer [20], prostate tumor [21], gastric cancer [22], breast cancer [23], lung carcinomas, etc. Overexpression of NRP-1 was found to promote tumor angiogenesis as well as the growth and proliferation of cancer cells. On the other hand, NRP-1 plays an important role in the immune system. NRP-1 expression in immune cells such as tumor-associated macrophages, T lymphocytes, and dendritic cells is related to tumor growth, migration, and cell‒cell interaction [24,25].

Metabolomics is a powerful approach that could reflect dynamic changes of small molecule metabolites when disturbance such as disease and drug-taking happens [26]. Since metabolites are located at the end of a biological information flow, changes could reflect the signal amplification effects of biochemical changes. Therefore, metabolomics could act as a tool to reveal the mechanism between TCM and potential targets.

In our present study, in order to explore new targets and reveal the in-depth mechanism of SSd, network pharmacology and metabolomics were used in combination. First, potential targets of SSd were obtained by database searching, literature mining, or chemical similarity predicting. Then molecular docking was performed to investigate the binding affinity of SSd with potential targets. Finally, untargeted metabolomics analysis was carried out to reveal the possible mechanism by which SSd affected the aimed target and the role of NRP-1.

## 2. Results

### 2.1. NRP-1 Was Selected as a Target of SSd

Fourteen targets of SSd were obtained from the HIT [10] and TCMID [11] databases (Table S1). Furthermore, 19 proteins were collected by literature mining and 14 targets were filtered from Pubchem by chemical similarity predicting (Tables S1–S3) [27]. Targets obtained by the three methods overlapped in some cases, which proved the reliability of the methods, as shown in Figure S1. A total of 35 proteins were pooled as a target set (Table S4). Then SSd was docked with all the targets. Twenty proteins were found to bind with SSd and were ranked by docking scores (Table 1). Prostaglandin G/H synthase 2 (COX-2) showed the strongest interaction with SSd, followed by neuropilin-1 (NRP-1) and transcription factor p65. COX-2 was reported to inhibit DEN-induced liver cancer in rats by SSd [28]. Others found that SSd downregulated transcription factor p65 and mediated inflammatory signaling to protect mice from hepatotoxicity induced by acetaminophen (APAP) [29]. However, the interaction between SSd and NRP-1 was not reported. Thus, we selected NRP-1 for further study.

### 2.2. NRP-1 Knockdown Enhanced the Anti-Hepatoma Effect of SSd

**Figure 1:**
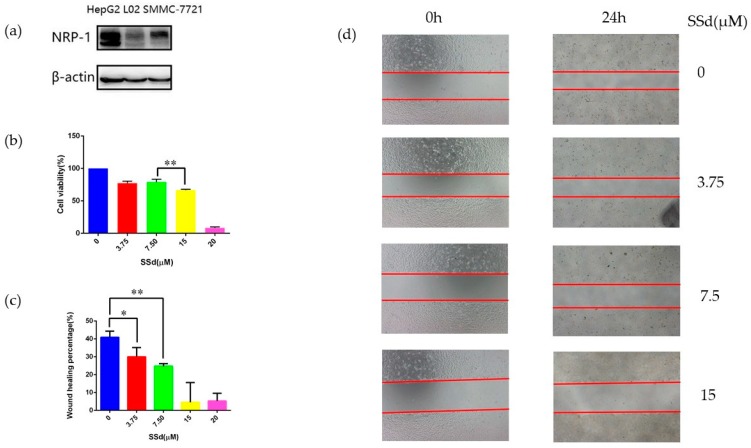
Cell lines comparison and the effects of SSd on HepG2. (**a**) Expression of NRP-1 in three cell lines; (**b**) cell viability of HepG2 after treatment with SSd, ** *p* < 0.01; (**c**) wound healing percentage of HepG2 after treatment with SSd, * *p* < 0.05, ** *p* < 0.01; (**d**) wound healing pictures of HepG2 after treatment with SSd.

NRP-1 has been reported to display higher expression in hepatocellular carcinoma cell lines than in normal hepatic cell lines [36]. In addition, the published data indicate that a tumor was suppressed by silencing NRP-1. Therefore, it was speculated that the anti-hepatoma effect of SSd might be enhanced if we could downregulate NRP-1 [37,38]. In order to select the most suitable cell lines, we compared NRP-1 expression levels in hepatocellular carcinoma cell lines HepG2 and SMMC-7721 and a normal hepatic cell line L-02 by Western blot (Figure 1a). High NRP-1 expression was detected in the HepG2 cell line, followed by SMMC-7721. L-02 showed the lowest expression of NRP-1, as reported [38]. Consequently, HepG2 was chosen for further study.

**Figure 2:**
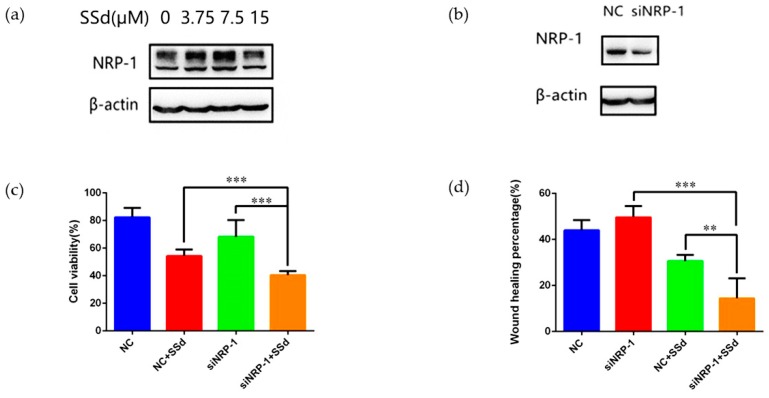
NRP-1 knockdown and effects of SSd on HepG2 in combination with NRP-1 knockdown. (**a**) The expression of NRP-1 affected by SSd; (**b**) knockdown of NRP-1; (**c**) the cell viability of HepG2 cells after knockdown of NRP-1 in combination with SSd, *** *p* < 0.001; (**d**) the wound healing percentage of HepG2 cells after knockdown of NRP-1 in combination with SSd, ** *p* < 0.01, *** *p* < 0.001.

Firstly, different concentrations of SSd were used to treat HepG2 and the IC_50_ was 16.02 ± 0.91 μM. The effect of SSd on migration of HepG2 was also measured, as shown in Figure 1c. The results demonstrated that SSd could inhibit the migration of HepG2. Next we investigated NRP-1 levels influenced by SSd. Upregulation of NRP-1 was observed when treated with 3.75 μM and 7.5 μM SSd. However, NRP-1 expression displayed no significant difference at 15 μM compared to the control, as shown in Figure 2a. According to the cell viability, HepG2 was damaged more severely at 15 μM SSd than at 7.5 μM or 3.75 μM, which suggested the mechanism at 15 μM or higher may not be directly related to NRP-1. So we selected a concentration of SSd at 7.5 μM for further investigation.

After NRP-1 was knocked down, HepG2 was treated with SSd. Then both cell viability and migration were measured. Compared to the siNRP-1 (small interfering RNA of gene NRP-1) and SSd-treated NC (negative control) groups, cell viability and migration were decreased significantly in the SSd-treated siNRP-1 group, as shown in Figure 2b–d. The results suggested that NRP-1 knockdown could significantly enhance the anti-hepatoma effect of SSd.

### 2.3. Quantitative Evaluation of the Effect of NRP-1 Knockdown and SSd by Metabolomics

**Figure 3:**
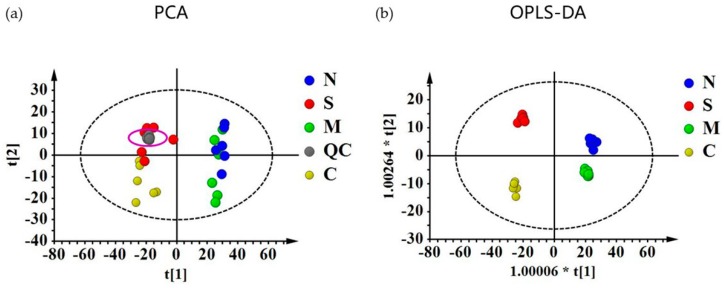
(**a**) Score plots of principal components analysis (PCA) and (**b**) orthogonal partial least squares discriminant analysis (OPLS-DA) models. C:NC samples, M:NC samples treated with SSd, S: siNRP-1 samples, N: siNRP-1 samples treated with SSd.

For all the metabolites obtained by GC/MS and LC/MS, PCA and OPLS-DA models were constructed, as shown in Figure 3. Results from GC/MS and LC/MS were all integrated into one PCA or OPLS-DA plot. In the PCA models, all the QC samples were clustered closely, which indicated a satisfactory analytical performance. The supervised OPLS-DA models showed clearly separation among the four groups and model statistics, R^2^X, R^2^Y and Q^2^ suggested the robustness of the models (Table S5).

**Figure 4:**
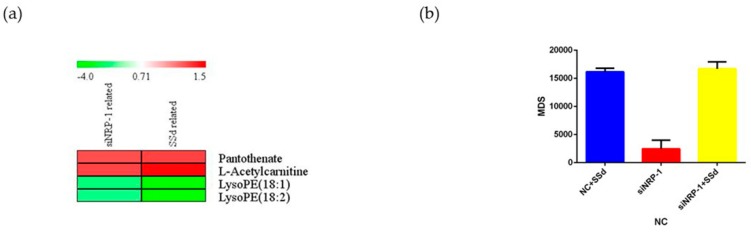
Fold changes of differential metabolites and MDS of all metabolites. (**a**) Heatmap of the fold changes of differential metabolites related to NRP-1 knockdown and SSd-treated; (**b**) MDS of all the metabolites detected with NC as control.

To further evaluate the influence of NRP-1 and SSd quantitatively, MDS was calculated. The results (Figure 4b) showed that the NRP-1 knockdown group displayed a smaller distance from the principal curve than the combination with SSd or SSd alone, which showed that the metabolic profile had a minor influence. On the other hand, a strong disturbance of the metabolic profile was demonstrated in the SSd-treated NC group as well as the SSd-treated siNRP-1 group.

### 2.4. Carnitines and Phospholipids Were Focalized Based on Metabolomics

**Figure 5:**
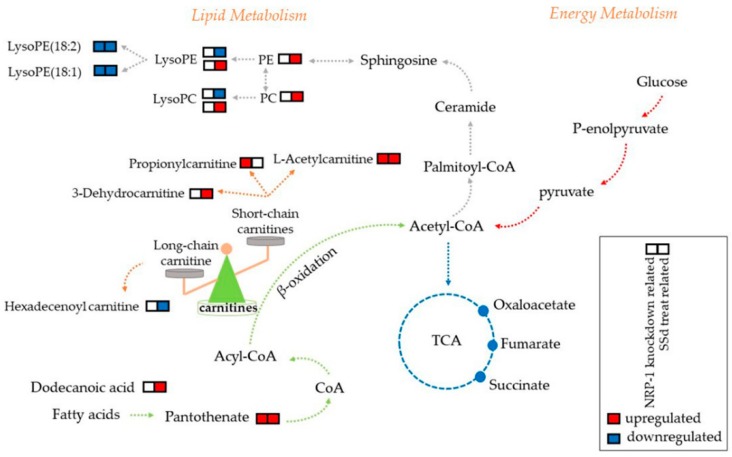
The possible perturbed metabolic pathways in response to NRP-1 knockdown and/or SSd. Upregulated (red) and downregulated (blue) levels of metabolites observed are indicated.

Variables with VIP > 1 and *p* < 0.05 were considered differential metabolites. For LC/MS, mass-to-charge (*m*/*z*) and MS/MS fragmentation patterns of metabolites were compared to the information provided in the HMDB database for annotation. Metabolites obtained by GC/MS were annotated by comparison with the National Institute of Standard and Technology library. Six differential metabolites were related to NRP-1 knockdown, 30 related to SSd-treated, and four appeared relevant to both. In general, NRP-1 knockdown combined with SSd treatment affected lyso-phosphatidylethanolamines, acetylcarnitine, and pantothenate. For those only affected by one factor, propionylcarnitine and meso-erythritol were related to NRP-1 knockdown, while hexadecenoylcarnitine, dodecanoic acid, galactose, 1-monooleoylglycerol, lyso-phosphatidylcholines, lyso-phosphatidylethanolamines, phosphatidylcholines and phosphatidylethanolamines were disturbed by SSd treatment. The fold changes of the differential metabolites are shown in Figure 4a. A hypothetical pathways investigation is shown in Figure 5.

## 3. Discussion

In our study, we collected targets and investigated the affinity among SSd and potential proteins. Results showed that COX-2, NRP-1, and transcription factor p65 were the top three based on docking scores, which suggested the existence of strong interactions with SSd. Cancer-related targets such as COX-2, transcription factor p65, cellular tumor antigen p53 [31], and myc proto-oncogene protein [32] have been reported to be influenced by SSd. Other reported inflammatory and apoptosis-related targets associated with SSd also gained satisfactory docking scores, which proved the reliability of the methods applied. However, up to now no research has been reported on the relationship between NRP-1 and SSd.

In our research, NRP-1 was found to be upregulated on the 3.75 μM and 7.5 μM of SSd, while the cell viability of HepG2 was maintained at approximately 75%. Overexpression of NRP-1 was reported to promote the proliferation of cancer cells [20], which probably suggested that the cell damage effect of 7.5 μM SSd was antagonized by the proliferation promotion of upregulating NRP-1. This leads us to ask whether NRP-1 knockdown would enhance the damage effect of SSd at 7.5 μM, due to the upregulated NRP-1 and unexpected side effects appearing simultaneously with the anti-hepatoma effect of SSd. After knockdown of NRP-1, the results showed that, compared to the siNRP-1- and NC-treated SSd groups, the cell viability and wound healing percentage of siNRP-1 treated with SSd decreased significantly, not counting the damage of the transfection reagent. The outcomes above have clarified that NRP-1 knockdown enhanced the cell damage of SSd. NRP-1 acts as a co-receptor of VEGF, which has been proven essential for the progression and metastasis for tumors [39]. However, studies showed that adding external VEGF did not have an impact on the apoptosis of cancer cells when NRP-1 was knocked down, which proved the crucial role of NRP-1 in the anti-tumor effect of VEGF/NRP-1 [40]. Our study provided evidence of the enhanced anti-hepatoma effect of SSd on hepatocellular carcinoma cells with NRP-1 knockdown. However, such a hypothesis still remains to be confirmed in vivo.

To further explore the possible mechanism, a metabolomics study was performed. PCA and OPLS-DA proved the overall difference between groups. MDS was calculated as an indicator to quantitively evaluate the extent of disturbance by NRP-1 knockdown and SSd on HepG2 [41,42,43]. The results showed that NRP-1 knockdown exhibited the lowest MDS, which demonstrated that the global metabolic profile would not be severely influenced. However, SSd treated alone or NRP-1 knockdown in combination with SSd groups had a significant influence. These results suggested that SSd would have a great influence on the metabolism of both untreated and NRP-1 knockdown cells, in accordance with the more differential metabolites related to SSd. Even though it was not demonstrated by MDS, the elevated damage effect of NRP-1 knockdown in combination with SSd on HepG2 has been verified by our in vitro study.

In our study, four annotated differential metabolites related to both NRP-1 knockdown and SSd treatment were observed to have the same trend during an intervention, which suggested that the combined action of those metabolites contributed to the advanced anti-hepatoma effect. There were also differential metabolites influenced by SSd or NRP-1 knockdown, respectively. We propose that combining the action of common and particular metabolites contributed to the intensive effect. The annotated differential metabolites mainly included short- and long-chain acetylcarnitines, pantothenate, lyso-phosphatidylethanolamine (LPE), lyso-phosphatidylcholines (LPC), phosphatidylethanolamine (PE), and phosphatidylcholines (PC). Thus, we analyzed the possible underlying mechanisms.

Among all the annotated metabolites, pantothenate and acetylcarnitine were related to both NRP-1 knockdown and SSd, a short-chain carnitine, propionylcarnitine was elevated by NRP-1 knockdown, while an intermediate in carnitine degradation, 3-dehydrocarnitine, and a long-chain carnitine, hexadecenoyl carnitine, were detected to be influenced by SSd. A short-chain carnitine, acetylcarnitine, increased in our study; however, the long-chain carnitine was decreased significantly. The elevation of 3-dehydrocarnitine was probably related to the degradation of long-chain carnitines [44]. Both short- and long-chain acetylcarnitines are known to play important roles in fatty acid metabolism. Long-chain acetylcarnitines are responsible for transporting long-chain fatty acids into mitochondria. Fatty acids are then oxidized and converted to energy by the TCA cycle [45,46]. The decrease in long-chain carnitines suggested a blockage of the energy production in cancer cells. For the excess short- and medium-chain fatty acids, acetylcarnitine was responsible for the removal from the mitochondria. The increased acetylcarnitine implied the accumulation of short- and medium-chain fatty acids. Others also reported that increased long-chain carnitine and decreased short-chain carnitine were observed in hepatocellular carcinoma [47]. Combined in our study, the increase of short-chain carnitines and decrease of the long-chain carnitine probably contribute to the enhanced damage to HepG2. However, such a hypothesis should be verified by further research.

Lyso-phosphatidylethanolamine (LPE), lyso-phosphatidylcholines (LPC), phosphatidylethanolamine (PE), and phosphatidylcholines (PC) were influenced by SSd treatment, while only LPE (18:1) and LPE (18:2) were observed to be downregulated by both NRP-1 knockdown and SSd treatment. Four LPEs decreased and one increased under the intervention of SSd. Three LPCs increased and two decreased after treatment with SSd, whereas all the PEs and PCs were increased. Those reflected the dysregulation in phospholipid metabolism. LPC is derived from PC, which is a component of cell membranes and mitochondrial membrane. Three methylation reactions can transform PE to PC [48]. The results showed that a change of LPE (18:1) and LPE (18:2) possibly contributed to the cell damage caused by NRP-1 knockdown and SSd, while disorder in phospholipid metabolism was mainly induced by SSd rather than NRP-1 knockdown. Out metabolomic studies indicated that the enhanced anti-hepatoma effect of NRP-1 knockdown, in combination with SSd, mainly influenced the lipid metabolism, but this should be validated in the future.

## 4. Materials and Methods

### 4.1. Chemicals and Reagents

Cell culture medium RPMI 1640, DMEM, penicillin, and streptomycin were purchased from Gibco (Carlsbad, CA, USA). Saikosaponin d (purity > 98%) was purchased from Sichuan Victory Biological Technology Co., Ltd. (Chengdu, China). Fetal bovine serum (FBS) was purchased from Biological Industries (Kibbutz Beit-Haemek, Israel). Cell Counting Kit-8 detection kit and BCA protein assay kit were obtained from Beyotime (Shanghai, China). PVDF membrane and chemiluminescence (ECL) reagent were purchased from Millipore (Burlington, MA, USA). The primary antibodies were obtained from Cell Signaling Technology (Boston, MA, USA) and HRP-conjugated secondary antibodies were purchased from Proteintech (Philadelphia, PA, USA). The siNRP-1 sequences were provided and synthesized by GenePharma (Shanghai, China). Lipofectamine 2000 reagent was purchased from Invitrogen (Carlsbad, CA, USA). Heptadecanoic acid, glibenclamide, methoxyamine hydrochloride, *N*-methyl-*N*-trifluoroacetamide (MSTFA) and pyridine were purchased from Sigma-Aldrich (St. Louis, MO, USA). Methanol and ethyl acetate (HPLC grade) were purchased from Merck (Darmstadt, Germany). Distilled water was purified by a Milli-Q system (Merckmillipore, Darmstadt, Germany).

### 4.2. Target Collection and Screening

#### 4.2.1. Target Collection

First, we searched several databases for the included SSd targets such as Traditional Chinese Medicine Systems Pharmacology Database and Analysis Platform (TCMSP version 2.3, http://lsp.nwu.edu.cn/tcmsp.php), Herbal Ingredients’ Targets Database (HIT, http://lifecenter.sgst.cn/hit/) and Traditional Chinese Medicine Integrative Database (TCMID, https://academic.oup.com/nar/article/41/D1/D1089/1057998). The keywords for database searching were “saikosaponin d.”

Secondly, reported targets of SSd were collected from literature mining. The keywords for literature mining were “saikosaponin d” and “targets.”

Lastly, we predicted targets by chemical similarity via the Pubchem resource (https://pubchem.ncbi.nlm.nih.gov/). Search entries of three databases, BioAssay, Compound, and Substance, can be reached on the homepage. Our protocols were in three main steps, as follows: (1) Structure download. The 2D structure formula of SSd was obtained via the Compound entry. (2) Selecting bioactive compounds. After uploading the *sdf* format of SSd in Identity/Similarity searching, similar scores were defined by 95%. Then, we filtered bioactive compounds of SSd and its structure analogs that have been identified via BioAssays, Active. (3) Tracking down potential targets. Targets of bioactive compounds were listed in Bioactivity Analysis. For all the targets, only those with more than one corresponding active compound can be subsequently collected as putative targets. Targets of SSd and its structural analogs could be obtained through this method.

For the targets achieved above, unified standard protein names and gene names were searched in the Uniprot (https://www.uniprot.org/) database. Then the target numbers from the three sources were input into Venny 2.1.0 (http://bioinfogp.cnb.csic.es/tools/venny/index.html) to view overlapping ones and test the reliability of the methods.

#### 4.2.2. Target Screening by Molecular Docking

To investigate the binding affinity of SSd and the candidate targets, molecular docking was carried out by Maestro 9.6 (Schrödinger, Inc, San Diego, CA, USA). First, the crystal structures of candidate proteins with high resolution and small ligands contained in the structures were downloaded from RCSB PDB (https://www.rcsb.org/). Next, we preprocessed the proteins by deleting the water molecules and other ligands. After energy minimizing, receptor-grid files and active sites were generated. Finally, SSd was docked at the active sites of the proteins after hydrogenating and charging. The targets were then sorted by docking scores and the degree of novelty.

### 4.3. Verification of the Target on HepG2

#### 4.3.1. Cell Culture

Human hepatocellular carcinoma cell line HepG2 was obtained from Type Culture Collection Cell Bank (Chinese Academy of Sciences Committee, Shanghai, China), human hepatocellular carcinoma cell lines SMMC-7721 and human hepatocyte L-02 were kindly provided by Prof. Yong Yang (Center for New Drug Safety Evaluation and Research, China Pharmaceutical University). SMMC-7721 and L-02 cell lines were cultured in RPMI 1640 with 10% FBS, 100 U/mL penicillin and 100 mg/mL streptomycin. HepG2 were cultured in DMEM with 10% FBS, 100 U/mL penicillin and 100 mg/mL streptomycin. All cells were cultured at 37 °C in a humidified incubator (Thermo, Waltham, MA, USA) with 5% CO_2_.

#### 4.3.2. Cell Proliferation Assay

The viability of HepG2 was measured by a Cell Counting Kit-8 (CCK-8) detection kit after treatment with different concentrations (1.75, 3.75, 7.5, 15 μM) of SSd. Briefly, 5 × 10^3^ cells per well were seeded in 96-well plates and treated with SSd. After 24 h treatment, CCK-8 was added and absorbance at 450 nm was measured after 2 h incubation at 37 °C.

#### 4.3.3. Cell Migration Assay

Migration of HepG2 was determined by a wound healing assay. The scratches were made after cell adherence in six-well plates. After rinsing with PBS, a medium containing different concentrations (1.75, 3.75, 7.5, 15 μM) of SSd and no FBS was replaced. Pictures obtained at 0 h and 24 h after scratching and distance of migration were analyzed by Image J (Version 1.48, National Institutes of Health, Bethesda, MD, USA).

#### 4.3.4. Western Blot

For the Western blot analysis of NRP-1 expression in blank HepG2, SMMC-7721 and L-02, protein levels of the three cell lines were determined by a BCA protein assay kit. Proteins were also measured for the Western blot analysis of NRP-1 expression in HepG2 after treatment with different concentrations of SSd. Thirty micrograms of protein per well were resolved by SDS-polyacrylamide gel, transferred to PVDF membrane, and blocked with 5% skim milk in phosphate-buffered saline containing 0.1% Tween 20. The membranes were incubated with primary antibodies at 4 °C overnight and probed with HRP-conjugated secondary antibodies at room temperature on the following day. The bands were visualized by Tanon (Shanghai, China) after using a chemiluminescence (ECL) reagent.

#### 4.3.5. Knockdown of NRP-1 Using Small Interfering RNA

HepG2 were cultured in a six-well plate then transfected with a mixture of Lipofectamine 2000 reagent and siRNAs in serum-free DMEM for 6 h. Cells were collected and we detected the knockdown efficiency by Western blot after incubating them for 48 h.

#### 4.3.6. Effect of SSd on siNRP-1 Cells

After transfection with siNRP-1/NC on HepG2 for 48 h, the medium was discarded and 7.5 μM SSd was treated for another 24 h. Cell viability and migration were then measured to evaluate the effect of NRP-1 knockdown in combination of SSd.

#### 4.3.7. Statistical Analysis

Data analyses were performed by SPSS 19.0 software (Chicago, IL, USA) and results were expressed as mean ± standard deviation (SD). The statistical significance of differences between the two groups was indicated by a Student’s *t* test. *p* < 0.05 was considered statistically significant.

### 4.4. Untargeted Metabolomics Analysis

#### 4.4.1. Sample Collecting and Metabolomic Analysis

HepG2 were seeded in culture plates and divided into four groups (siNRP-1 group, NC group, siNRP-1 and SSd-treated group, NC and SSd-treated group, *n* = 6). Cells were treated as described above. Culture medium containing SSd was removed before 3 mL cold methanol was added. Internal standard was dissolved in methanol firstly, 5 μg/mL heptadecanoic acid for GC, and 20 μg/mL glibenclamide for LC. Then the samples were quenched at −80 °C for 20 min. Cells were scraped, ultrasonicated for 3 min, and centrifuged for 10 min to collect the supernatant for subsequent procedures. We dried the extracts with nitrogen flow at 37 °C and redissolved them with methanol before metabolomic analysis. Sample preparation and methods of instruments were based on our previous studies [49,50,51], as shown in the Supplementary Materials.

GC-MS analysis was performed on Shimadzu GCMS-QP2010 Ultra (Ultra GC-Q/MS; Shimadzu Inc., Kyoto, Japan) equipped with an Rtx-5MS capillary column (30.0 m × 0.25 mm ID, 0.25 μm). LC-MS analysis was carried out on a Shimadzu Prominence series ultrafast liquid chromatography (UFLC) system coupled with an ion trap time-of flight mass spectrometry system (IT-TOF/MS) (Shimadzu Inc.). Separation was achieved by a Phenomenex Kinetex C18 column (100 mm × 2.1 mm, 2.6 μm) (Phenomenex, Torrance, CA, USA).

#### 4.4.2. Data Preprocessing and Analysis

Data preprocessing and analysis were also based on our previous studies [52,53]. The original chromatogram was processed for peak deconvolution and alignment by Profiling Solution (Shimadzu, Kyoto, Japan, version 1.1). The primary parameters were set as follows: ion *m*/*z* tolerance (500 mDa for GC/MS and 20 mDa for LC/MS), ion retention time tolerance (0.1 min for GC/MS and 0.2 min for LC/MS), and ion intensity threshold (10,000 counts for GC/MS and 8000 counts for LC/MS). The data were then exported to Excel and handled according to the “80%” rule: only metabolites detected in at least 80% of one group or more would be kept. Variables with relative standard deviation (RSD) lower than 30% in quality control (QC) samples were retained for further analysis.

The preprocessed data were imported into SIMCA-P (Umetrics, Sweden, Version 13.0). After pareto scaling of all the variables, principal components analysis (PCA) and orthogonal partial least squares discriminant analysis (OPLS-DA) were performed. The normalization by total ion intensity was performed to standardize multivariant analysis. Differences among groups could be observed. The differential metabolites were selected by variable importance in the projection (VIP) and *p*-values obtained by Mann‒Whitney U test, variables with VIP > 1 and *p* < 0.05 were screened out.

#### 4.4.3. Quantitative Evaluation of NRP-1 Knockdown and SSd on HepG2 by Metabolites Deregulation Score (MDS)

Effects of NRP-1 knockdown and SSd treatment could be quantified by the metabolites deregulation score (MDS). All the metabolites detected were integrated as a whole dataset. Then the R package pathifier was applied to analyze the metabolite sets. The whole dataset was considered as a cloud and we built a “principal curve” through the NC samples in the cloud [44]. Since untargeted metabolomic method covers limited metabolic pathways, we calculated the overall MDS instead of the pathway deregulation score (PDS) by the R package pathifier [45,46]. Each sample was projected onto the principal curve and the projection distance was MDS.

#### 4.4.4. Differential Metabolites Annotation

Annotation of metabolites detected by GC/MS was carried out by comparison with the National Institute of Standard and Technology library. Peaks with more than 80% similarity were assigned corresponding compound names. For LC/MS, mass-to-charge (*m*/*z*) and MS/MS fragmentation patterns of metabolites were compared with the information provided in the HMDB database (http://www.hmdb.ca/) for annotation. Then, retention time, accurate *m*/*z*, and the MS/MS fragmentation of features of interest were compared with those standard compounds available in our lab. Pathways by which metabolites got involved were searched and enriched on the MetaboAnalyst (version 4.0) and KEGG databases (https://www.kegg.jp/).

For the differential metabolites, NRP-1 knockdown and SSd-related were filtered to calculate the fold change of concentrations.

## 5. Conclusions

In our study, potential targets of SSd were predicted and NRP-1 was chosen for further research. Experimental proof of the enhanced anti-hepatoma effect of SSd in combination with NRP-1 knockdown was provided for the first time. Further metabolomics study revealed that lipid transportation and phospholipid metabolism were significantly altered when NRP-1 knockdown and SSd were treated on HepG2. Our findings indicate a new insight into the understanding of mechanism of SSd, but this should be confirmed in the future.

## Figures and Tables

**Table 1:** Docking results of SSd and potential targets.

Number	PDB ID	Gene Name	Uniprot ID	Target Name	Docking Score	Validated or Not
1	5F19	PTGS2	P35354	Prostaglandin G/H synthase 2	−6.67	Validated [28]
2	2QQI	NRP1	O14786	Neuropilin−1	−6.469	Unvalidated
3	1NFI	RELA	Q04206	Transcription factor p65	−5.585	Validated [1]
4	2W96	CDK4	P11802	Cell division protein kinase 4	−5.577	Validated [30]
5	1IKN	NFKBIA	P25963	NF-kappa-B inhibitor alpha	−5.346	Validated [1]
6	5JFD	F2	P00734	Prothrombin	−5.325	Unvalidated
7	4AGN	P53	P04637	Cellular tumor antigen p53	−5.205	Validated [31]
8	4MAN	BCL-2	P10415	Apoptosis regulator Bcl-2	−4.857	Validated [7]
9	5TBE	MAPK14	Q16539	Mitogen-activated protein kinase 14	−4.792	Validated [32]
10	1IL6	IL6	P05231	Interleukin-6	−4.666	Validated [1]
11	3V3K	CASP9	P55211	Caspase-9	−4.49	Validated [8]
12	4PRY	CASP3	P42574	Caspase-3	−4.443	Validated [8]
13	5T46	EIF4G1	Q04637	Eukaryotic translation initiation factor 4	−4.414	Unvalidated
14	4LXO	FN1	P02751	Fibronectin	−4.303	Unvalidated
15	2DBF	NFKB1	P19838	Nuclear factor NF-kappa-B p105	−3.902	Unvalidated
16	5FF0	TGFB1	P01137	Transforming growth factor beta-1	−3.642	Validated [33]
17	5T01	JUN	P05412	Transcription factor AP-1	−3.611	Validated [32]
18	4S0O	BAX	Q07812	Apoptosis regulator BAX	−3.412	Validated [8]
19	4FDL	CASP7	P55210	Caspase-7	−3.328	Validated [34]
20	5I4Z	MYC	P01106	Myc proto-oncogene protein	−2.997	Validated [35]

## Supplementary Materials

The following are available online: Figure S1: The intersection of targets amount obtained from three sources; Figure S2: The pictures of cell migration assay after NRP-1 knockdown and/or SSd treatment; Table S1: Potential targets of SSd searched by databases; Table S2: Targets obtained by literature mining; Table S3: Targets and its active compounds predicted by SSd and its structural analogues in Pubchem; Table S4: All the predicted targets of SSd; Table S5: Statistical parameters of the models; Table S6: Differential metabolites relevant to both NRP-1 knockdown and SSd treatment; Table S7: Differential metabolites related to NRP-1 knockdown; Table S8: Differential metabolites related to SSd treated.
