# Supplementary material for: Untargeted Metabolomics Study of the In Vitro Anti-Hepatoma Effect of Saikosaponin d in Combination with NRP-1 Knockdown
Source: Molecules. 2019 Apr 11;24(7):1423. doi: 10.3390/molecules24071423 (PMC6480384; doi:10.3390/molecules24071423)
Supplement: Supplementary file 1 [file molecules-24-01423-s001.pdf]

Untargeted Metabolomics Study of the In Vitro Anti-Hepatoma Effect of Saikosaponin d in Combination with NRP-1 Knockdown

Yingtong Lv [1,2], Xiaoying Hou[1,2], Qianqian Zhang[1,2], Ruiting Li[1,2], Lei Xu[1,2], Yadong Chen[3], Yuan Tian[1,2], Rong Sun[4], Zunjian Zhang[1,2,*] and Fengguo Xu[1,2,*]

[1] Key Laboratory of Drug Quality Control and Pharmacovigilance (Ministry of Education), State Key Laboratory of Natural Medicine, China Pharmaceutical University, Nanjing 210009, China; lv_yingtong@126.com (Y.L.); cpuhxy_2011@163.com (X.H.); zhangqianqian09413@163.com (Q.Z.); 15051853218@163.com (R.L.); 18356002797@163.com (L.X.); 1020041334@cpu.edu.cn (Y.T.)

[2] Jiangsu Key Laboratory of Drug Screening, China Pharmaceutical University, Nanjing 210009, China

[3] Department of Organic Chemistry, China Pharmaceutical University, Nanjing 210009, China; ydchen@cpu.edu.cn

[4] Advanced Medical Research Institute, Shandong University, Jinan 250100, China; sunrong107@163.com

* Correspondence: zzj@cpu.edu.cn (Z.Z.); fengguoxu@cpu.edu.cn (F.X.); Tel.: +86-25-83271021 (F.X.)

Methods

Sample preparation and instrumental analysis for metabolomic were based on our previous studies [49-51].

1. Sample preparation for metabolomic analysis

After drying the extracts with nitrogen flow, 120μL methanol was added to redissolve and centrifuge at 4℃, 16000rpm for 10 minutes twice.

For GC-MS analysis, 80μL supernatant was transferred to brown glass vials followed by addition of 25μL *O*-methoxyamine hydrochloride (10mg/ml in pyridine). Then the mixture was incubated for 90min at 37℃ and evaporated to dry for 2h at 50℃ by CentriVap Centrifugal Concentrator(Labconco, Kansas, MO, USA). 120μL *N*-methyl-*N*-trifluoroacetamide/ethyl acetate(1:4) was added and incubated for 2h at 37℃ then transferred the supernatant for GC-MS analysis.

For LC-MS analysis, 80μL supernatant was analyzed.

2. Instrumental Analysis

Liquid chromatography-mass spectrometry analysis was performed on LC-ion trap time-of flight MS system equipped with an electrospray ionization source (Shimadzu, Kyoto, Japan). Chromatographic separation was achieved on a Phenomenex Kinetex C_{18} column(100 mm×2.1 mm, 2.6μM, Phenomenex, USA). Samples were separated with gradient elution involved in a mobile phase consisting of (A)0.1% formic acid in water and (B)methanol at a flow rate of 0.4mL/min. The elution program was from 95% A to 0% A within 30 minutes and held for 3 minutes. The column oven was 40℃ and the injection volume was 5 μL. Both positive and negative ion mode were acquired by switching the interface voltage between 4.5 and -3.5 kV in a full-scan operation with a scan range of mass to charge ratio 100 to 1000. The flow rate of nebulizing gas(N_2) was 1.5 L/min and pressure of drying gas was 100 kPa. The temperature of heat block and curved desorption line were both 200℃. LCMS solution software (Shimadzu, Kyoto, Japan) was used for mass spectra acquisition and chromatograms processing.

The gas chromatography analysis was performed on the GC-MS QP2010 Ultra

(Shimadzu Co., Kyoto, Japan) equipped with a fused silica capillary column (Rtx-5MS; 30m × 0.25mm i.d., film thickness 0.25μm, Restek, USA). Helium was applied as carrier gas at a flow rate of 1ml/min. Sample injection volume was 1μL with a split ratio of 20:1. The oven temperature was initially kept at 70℃ for 3min then increased at rate of 10℃/min to 320℃ and held for 2min. The temperature of injector, interface, and ion source were set at 250℃, 200℃, and 250℃, respectively. Ions were acquired at full scan mode with mass to charge ratio range from 45 to 600. Mass spectra and chromatograms were acquired and processed with GC-MS solution version 2.7 (Shimadzu, Kyoto, Japan).

Table S1. Potential targets of SSd searched by databases

Uniprot ID	Protein name	Gene name	Database
P02751	Fibronectin	FN1	HIT
P01137	Transforming growth factor beta-1	TGFB1	
P11802	Cell division protein kinase 4	CDK4	
P05412	Transcription factor AP-1	JUN	
P01100	Proto-oncogene c-Fos	FOS	
P01375	Tumor necrosis factor	TNF	
P05231	Interleukin-6	IL6	
Q04206	Transcription factor p65	RELA	
P25963	NF-kappa-B inhibitor alpha	NFKBIA	
P01106	Myc proto-oncogene protein	MYC	
P04150	Glucocorticoid receptor	NR3C1	
P10415	Apoptosis regulator Bcl-2	BCL2	
P38936	Cyclin-dependent kinase inhibitor 1	CDKN1A	TCMID
P04637	Cellular tumor antigen p53	TP53	

Table S2. Targets obtained by literature mining

Uniprot ID	Protein name	Gene name	
P25963	NF-kappa-B inhibitor alpha	NFKBIA	Dang et al. 2007[1]
P40763	Signal transducer and activator of transcription 3	STAT3	Liu et al.2014[29]
P05231	Interleukin-6	IL6	Dang et al. 2007[1]
Q04206	Transcription factor p65	RELA	Dang et al. 2007[1],Wong et al.2013[52]
P35354	Prostaglandin G/H synthase 2	PTGS2	Lu et al.2012[35]
P01375	Tumor necrosis factor	TNF	Dang et al. 2007[1],Wong et al.2013[52]
Q16665	Hypoxia-inducible factor 1-alpha	HIF1A	He et al.2014[53]
P42574	Caspase-3	CASP3	Chen et al.2016[8], Chiang et al.2003[34]
P55210	Caspase-7	CASP7	Chiang et al.2003[34]
P55211	Caspase-9	CASP9	Chen et al.2016[8]
P10415	Apoptosis regulator Bcl-2	BCL2	Zhang et al.2016[7],Hsu et al.2000[35]
Q07812	Apoptosis regulator BAX	BAX	Zhang et al.2016[7]
P04150	Glucocorticoid receptor	NR3C1	Li et al.2014[5]
P04637	Cellular tumor antigen p53	TP53	Hsu et al.2004[31],Hsu et al.2000[35]
P01106	Myc proto-oncogene protein	MYC	Hsu et al.2000[35]

P28482	Mitogen-activated protein kinase 1	MAPK1	Lin et al.2016[32]
P05412	Transcription factor AP-1	JUN	Lin et al.2016[32]
Q16539	Mitogen-activated protein kinase 14	MAPK14	Lin et al.2016[32]
P01137	Transforming growth factor beta-1	TGFB1	Fan et al.2007[33]

1. Shuang-Suo Dang; Bao-Feng Wang; Yan-an Cheng; Ping Song; Zhen-Guo Liu; Li., Z.-F. Inhibitory effects of saikosaponin-d on CCl4-induced hepatic fbrogenesis in rats. *World Journal of Gastroenterology* **2007**, *13*, 557-563.
5. Li, Z.Y.; Jiang, Y.M.; Liu, Y.M.; Guo, Z.; Shen, S.N.; Liu, X.M.; Pan, R.L. Saikosaponin D acts against corticosterone-induced apoptosis via regulation of mitochondrial GR translocation and a GR-dependent pathway. *Progress in Neuropsychopharmacology & Biological Psychiatry* **2014**, *53*, 80-89.
7. Zhang, F.; Chen, L.; Jin, H.; Shao, J.; Wu, L.; Lu, Y.; Zheng, S. Activation of Fas death receptor pathway and Bid in hepatocytes is involved in saikosaponin D induction of hepatotoxicity. *Environ. Toxicol. Pharmacol.* **2016**, *41*, 8-13.
8. Chen, M.F.; Huang, S.J.; Huang, C.C.; Liu, P.S.; Lin, K.I.; Liu, C.W.; Hsieh, W.C.; Shiu, L.Y.; Chen, C.H. Saikosaponin d induces cell death through caspase-3-dependent, caspase-3-independent and mitochondrial pathways in mammalian hepatic stellate cells. *BMC Cancer* **2016**, *16*, 532.
28. Lu, X.L.; He, S.X.; Ren, M.D.; Wang, Y.L.; Zhang, Y.X.; Liu, E.Q. Chemopreventive effect of saikosaponin-d on diethylinitrosamine-induced hepatocarcinogenesis: involvement of CCAAT/enhancer binding protein β and cyclooxygenase-2. *Molecular Medicine Reports* **2012**, *5*, 637-644.
29. Liu, A.; Tanaka, N.; Sun, L.; Guo, B.; Kim, J.H.; Krausz, K.W.; Fang, Z.; Jiang, C.; Yang, J.; Gonzalez, F.J. Saikosaponin d protects against acetaminophen-induced hepatotoxicity by inhibiting NF-κB and STAT3 signaling. *Chemico-Biological Interactions* **2014**, *223*, 80-86.
31. Hsu, Y.L.; Kuo, P.L.; Lin, C.C. The proliferative inhibition and apoptotic mechanism of Saikosaponin D in human non-small cell lung cancer A549 cells. *Life Sci.* **2004**, *75*, 1231-1242.
32. Lin, X.; Wu, S.; Wang, Q.; Shi, Y.; Liu, G.; Zhi, J.; Wang, F. Saikosaponin-D Reduces H 2 O 2 -Induced PC12 Cell Apoptosis by Removing ROS and Blocking MAPK-Dependent Oxidative Damage. *Cellular & Molecular Neurobiology* **2016**, *36*, 1-11
33. Fan, J.; Li, X.; Li, P.; Li, N.; Wang, T.; Shen, H.; Siow, Y.; Choy, P.; Gong, Y. Saikosaponin-d attenuates the development of liver fibrosis by preventing hepatocyte injury. *Biochem Cell Biol.***2007**, *85*, 189-195
34. Chiang, L.C.; Ng LTLiu, L.T.; Shieh, D.E.; Lin, C.C. Cytotoxicity and anti-hepatitis B virus activities of saikosaponins from bupleurum species. *Planta Medica* **2003**, *69*, 705-709.
35. Hsu, M.J.; Cheng, J.S.; Huang, H.C. Effect of saikosaponin, a triterpene saponin, on apoptosis in lymphocytes: association with c-myc, p53, and bcl-2 mRNA. *Br. J. Pharmacol.* **2000**, *131*, 1285-1293

52. Wong, V.K.W.; Molly Miao, Z.; Hua, Z.; Lam, K.Y.C.; Po Ling, C.; Law, C.K.M.; Yue, P.Y.K.; Liang, L. Saikosaponin-d Enhances the Anticancer Potencyof TNF-α via Overcoming Its Undesirable Response of Activating NF-Kappa B Signalling in Cancer Cells. *Evidence-Based Complementray and Alternative Medicine* **2013** 745295
53. He, S.; Lu, G.; Hou, H.; Zhao, Z.; Zhu, Z.; Lu, X.; Chen, J.;Wang, Z. Saikosaponin-d suppresses the expression of cyclooxygenase-2 through the phospho-signal transducer and activator of transcription 3/hypoxia-inducible factor-1α pathway in hepatocellular carcinoma cells. *Molecular Medicine Reports* **2014**, *10*, 2556.

Table S3. Targets and its active compounds predicted by SSd and its structural analogues in Pubchem

Protein targets	active compounds
Electroneutral potassium-chloride cotransporter KCC2	2
Regulator of G-protein signaling 4 isoform 2	2
Thyrotropin-releasing hormone receptor	2
D(2) dopamine receptor isoform long	2
D(1A) dopamine receptor	2
Eukaryotic translation initiation factor 4 gamma 1 isoform 4	1
Chain A, Crystal Structure Of The B1b2 Domains From Human Neuropilin-1	1
Mitogen-activated protein kinase 1	1
Corticotropin-releasing hormone receptor 2	1
Nuclear factor NF-kappa-B p105 subunit isoform 1	1
Chain A, Human Ape1 Endonuclease With Bound Abasic Dna And Mn2+ Ion	1
Prothrombin	1
Corticotropin releasing factor-binding protein sapiens	1
Myc proto-oncogene protein	1

Table S4. All the predicted targets of SSd

Number	Uniprot ID	Target name	Gene name
1	P02751	Fibronectin	FN1
2	P01137	Transforming growth factor beta-1	TGFB1
3	P11802	Cell division protein kinase 4	CDK4
4	P05412	Transcription factor AP-1	JUN
5	P01100	Proto-oncogene c-Fos	FOS
6	P01375	Tumor necrosis factor	TNF
7	P05231	Interleukin-6	IL6
8	Q04206	Transcription factor p65	RELA
9	P25963	NF-kappa-B inhibitor alpha	NFKBIA
10	P01106	Myc proto-oncogene protein	MYC
11	P04150	Glucocorticoid receptor	NR3C1
12	P10415	Apoptosis regulator Bcl-2	BCL2
13	P38936	Cyclin-dependent kinase inhibitor 1	CDKN1A
14	P04637	Cellular tumor antigen p53	TP53

15	P40763	Signal transducer and activator of transcription 3	STAT3
16	P35354	Prostaglandin G/H synthase 2	PTGS2
17	Q16665	Hypoxia-inducible factor 1-alpha	HIF1A
18	P42574	Caspase-3	CASP3
19	P55210	Caspase-7	CASP7
20	P55211	Caspase-9	CASP9
21	Q07812	Apoptosis regulator BAX	BAX
22	Q16539	Mitogen-activated protein kinase 14	MAPK14
23	Q9H2X9	Electroneutral potassium-chloride cotransporter KCC2	SLC12A5
24	P49798	Regulator of G-protein signaling 4	RGS4
25	P34981	Thyrotropin-releasing hormone receptor	TRHR
26	P14416	D(2) dopamine receptor	DRD2
27	P21728	D(1A) dopamine receptor	DRD1
28	Q04637	Eukaryotic translation initiation factor 4	EIF4G1
29	O14786	Neuropilin-1	NRP1
30	P28482	Mitogen-activated protein kinase 1	MAPK1
31	Q13324	Corticotropin-releasing hormone receptor 2	CRHR2

32	P19838	Nuclear factor NF-kappa-B p105	NFKB1
33	P27695	Ape1 Endonuclease	APEX1
34	P00734	Prothrombin	F2
35	P24387	Corticotropin releasing factor-binding protein	CRHBP

Table S5. Statistical parameters of the models

Model	R^2X	R^2Y	Q^2
PCA	0.751		0.508
OPLS-DA	0.71	0.963	0.71

Table S6. Differential metabolites relevant to both NRP-1 knockdown and SSd treated

No	Compound Name
1	Pantothenate
2	L-Acetylcarnitine
3	LysoPE(18:1)
4	LysoPE(18:2)

Table S7. Differential metabolites related to NRP-1 knockdown

Number	Compound Name	t_R/min	Ion(*m*/*z*)	Fold change
1	L-Acetylcarnitine	0.893	204.1212	1.28
2	Propionylcarnitine	1.119	218.1361	1.62
3	Pantothenate	2.39	220.1165	1.25
4	LysoPE(18:2)	19.734	500.2739	-1.42
5	LysoPE(18:1)	21.024	480.3084	-1.63
6	meso-Erythritol	13.179	222.5757	1.31

Table S8. Differential metabolites related to SSd treated

Number	Compound name	t_R/min	Ion(*m*/*z*)	Fold change
1	L-Acetylcarnitine	0.893	204.1212	1.45
2	3-Dehydrocarnitine	1.213	182.0799	1.41
3	Pantothenate	2.39	220.1165	1.28
4	Hexadecenoyl carnitine	17.72	398.325	-7.25
5	LysoPE(18:2)	18.697	500.2758	-3.32
6	LysoPC(16:1)	19.262	516.3049	2.39
7	LysoPE(18:1)	19.52	502.2917	-3.84
8	LysoPE(20:4)	19.969	502.2927	5.55
9	LysoPE(18:0)	20.25	504.3022	-13.44
10	LysoPE(16:0)	20.293	454.292	-21.26
11	LysoPC(16:0)	20.718	496.3399	1.40
12	LysoPC(18:1)	21.198	522.3547	1.41
13	LysoPC(15:0)	22.432	482.3295	-7.27
14	LysoPC(18:0)	22.57	524.3712	-10.58
15	PE(15:0/22:5)	28.402	752.5182	17.49
16	PE(15:0/20:3)	28.928	728.5201	4.58
17	PC(18:4/18:1)	29.072	780.5507	-11.10
18	PE(15:0/22:1)	29.406	782.5605	3.07
19	PE(15:0/18:3)	29.658	698.4854	6.76
20	PC(14:0/P-18:0)	29.951	718.5685	11.16
21	PE(20:3/15:0)	30.271	726.5171	1.87
22	PC(16:1/P-18:1)	30.372	742.568	2.00
23	PE(24:1/15:0)	30.762	788.6153	1.31
24	PC(22:4/16:0)	30.775	810.5964	1.60
25	PC(P-18:1/20:3)	30.818	794.6028	14.20
26	PC(20:1/20:4)	30.952	836.6137	3.72
27	PC(18:0/P-18:0)	31.235	796.6173	1.52

28	Dodecanoic acid	14.809	140.9265	2.06
29	Galactose	19.435	166.3328	1.66
30	1-Monooleoylglycerol	25.277	212.1432	1.44

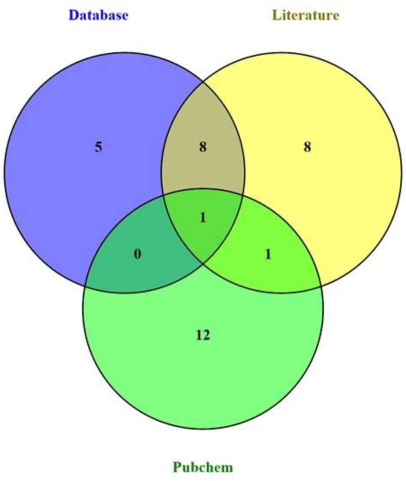

Figure S1. The intersections of targets amount from three sources

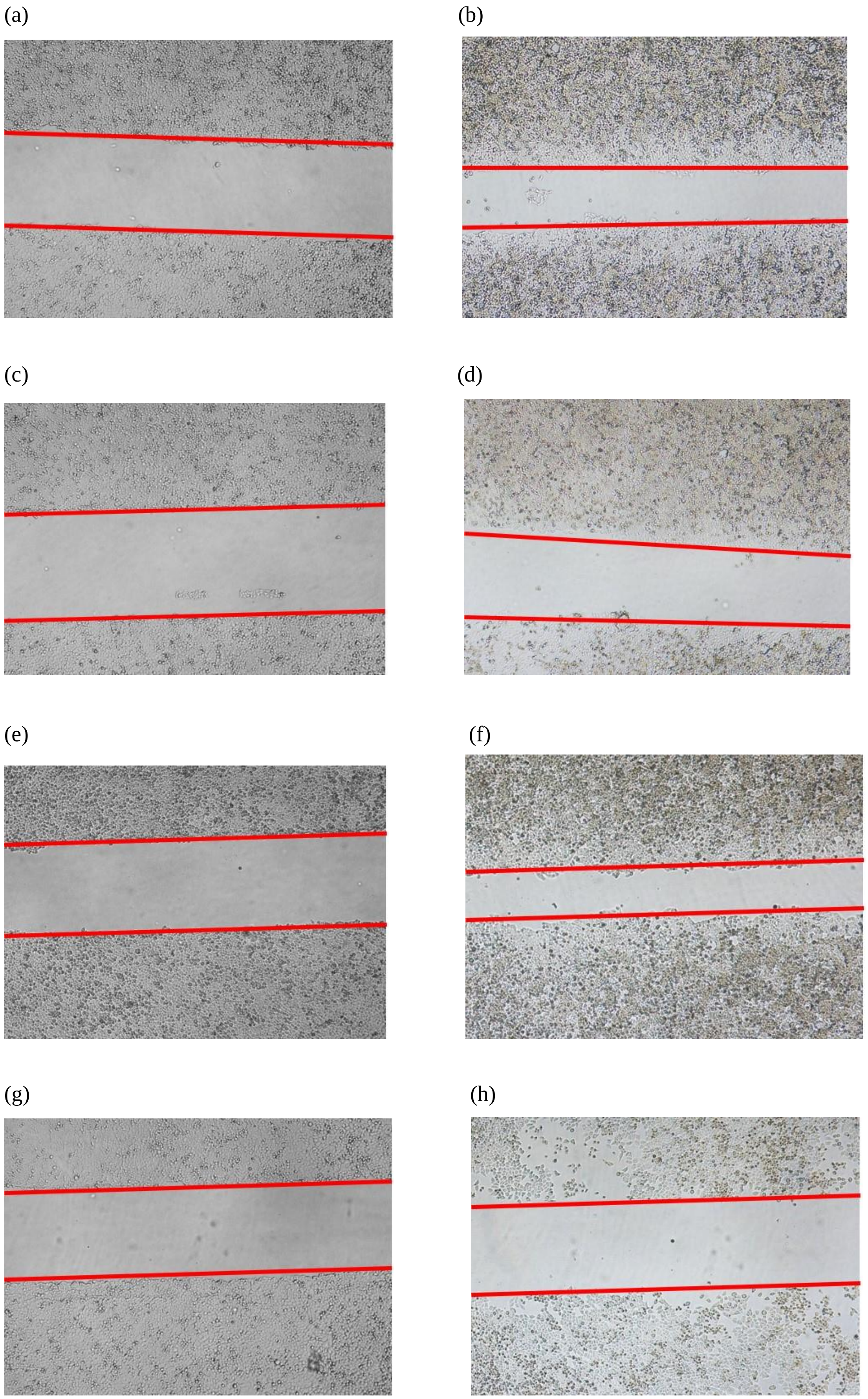
(a)
(b)
(c)
(d)
(e)
(f)
(g)
(h)

Figure S2. The pictures of cell migration assay after NRP-1 knockdown and/or SSd treated. (a) 0h after blank cell culture medium was treated on NC cells; (b) 24h after blank cell culture medium was treated on NC cells; (c) 0h after SSd was treated on NC cells; (d) 24h after SSd was treated on NC cells; (e) 0h after blank cell culture medium was treated on siNRP-1 cells; (f) 24h after blank cell culture medium was treated on siNRP-1 cells; (g) 0h after SSd was treated on siNRP-1 cells; (h) 24h after SSd was treated on siNRP-1 cells.
